# The CCN1 (CYR61) protein promotes skin growth by enhancing epithelial‐mesenchymal transition during skin expansion

**DOI:** 10.1111/jcmm.14828

**Published:** 2019-12-11

**Authors:** Yiwen Zhou, Haizhou Li, Xiao Liang, Hengyu Du, Yingjun Suo, Hao Chen, Wenhui Liu, Ran Duan, Xiaolu Huang, Qingfeng Li

**Affiliations:** ^1^ Department of Plastic & Reconstructive Surgery Shanghai Ninth People's Hospital Shanghai Jiao Tong University School of Medicine Shanghai China; ^2^ Institute of Biomedical and Pharmaceutical Sciences Guangdong University of Technology Guangzhou Higher Education Mega Center Guangzhou China

**Keywords:** CCN1, CYR61, epithelial‐mesenchymal transition, skin expansion, skin growth

## Abstract

The skin expansion technique is widely used to induce skin growth for large‐scale skin deformity reconstruction. However, the capacity for skin expansion is limited and searching for ways to improve the expansion efficiency is a challenge. In this study, we aimed to explore the possible mechanism of skin expansion and to find a potential therapeutic target on promoting skin growth. We conducted weighted gene coexpression network analysis (WGCNA) of microarray data generated from rat skin expansion and found CCN1 (CYR61) to be the central hub gene related to epithelial‐mesenchymal transition (EMT). CCN1 up‐regulation was confirmed in human and rat expanded skin and also in mechanically stretched rat keratinocytes, together with acquired mesenchymal phenotype. After CCN1 stimulation on keratinocytes, cell proliferation was promoted and partial EMT was induced by activating β‐catenin pathway. Treatment of CCN1 protein could significantly increase the flap thickness, improve the blood supply and restore the structure in a rat model of skin expansion, whereas inhibition of CCN1 through shRNA interference could dramatically reduce the efficiency of skin expansion. Our findings demonstrate that CCN1 plays a crucial role in skin expansion and that CCN1 may serve as a potential therapeutic target to promote skin growth and improve the efficiency of skin expansion.

## 1 INTRODUCTION

Skin expansion is a surgical technique that is widely applied in clinical situation for critical‐sized skin defect reconstruction of post‐burn or post‐trauma scars, congenital deformities and tumour resection.^1^, ^2^ This method utilizes a balloon‐like silicone expander to load continuous mechanical stretch onto the local skin to promote skin growth.^1^, ^3^ The additional skin tissue obtained by skin expansion is an ideal match for the skin defect due to its similarity in colour, texture and structure.^4^ Although skin expansion has been recognized as a reliable reconstructive method, it is not perfect. On the one hand, the growth capacity of skin in response to stretching is limited (usually less than 2‐3 times the original area).^5^, ^6^ Over‐expansion usually results in complications such as flap thinness, infection, ischaemia and even necrosis.^7^, ^8^ On the other hand, the current expansion efficiency is relatively low, and the skin expansion process usually requires several months.^9^ Hence, the identification of ways to improve the skin growth efficiency and generate sufficient functional skin tissue is a major challenge facing surgeons and researchers.

The basic principle of skin expansion is that mechanical stretch initiates a cascade of events that ultimately results in skin growth. The histological structure and mechanical properties of the newly grown skin are found to be consistent with the original skin.^6^ In the recent years, various studies have reported the possible mechanisms related to the skin growth during skin expansion^10^, ^11^ including increased mitotic activity in the epidermis and dermis^12^, ^13^ and elevated growth factors release (eg EGF, bGFG, PDGF and TGF‐β).^14^, ^15^ Nevertheless, the exact mechanism by which mechanical stretch influences skin biology remains unclear.

Epithelial‐mesenchymal transition (EMT) is a process during which epithelial cells lose their cell polarity and cell‐cell adhesion but gain mesenchymal characteristics including migratory and invasive abilities.^16^ EMT is essential for numerous developmental events, tissue regeneration, wound healing, tissue fibrosis and the metastasis of cancer cells.^17^ In some of the EMT process, cells that originate from epithelial cells exhibit both epithelial and mesenchymal phenotype, known as partial EMT. Cells undergo partial EMT are thought to acquire stemness traits and can differentiate into various cell types.^18^ It is reported that EMT has been shown to be induced after the application of biomechanical force.^19^, ^20^ Our previous work first found the existence of EMT in mouse‐expanded skin tissue^19^ and indicated that EMT is a crucial process in skin growth under mechanical stretch.

In the current study, we aimed to explore the possible mechanism of skin expansion and identify a potential therapeutic target for promoting skin growth. First, we established a rat model of skin expansion and collected the normal and expanded skin tissues for microarray analysis. Subsequently, we used WGCNA to locate the central hub gene that is most related to the EMT during skin expansion. Then, we performed multiple in vitro and in vivo experiments to demonstrate the effect of the central hub gene on skin expansion and its underlying mechanism.

## 2 METHODS

### 2.1 Collection of human samples and histology

The expanded skin (ES) and adjacent normal skin (NS) specimens were collected from faciocervical region of the patients (10 donors in total; average age 26.9 ± 7.4 years old) who underwent reconstructive surgery using a skin expansion method. Written informed consent was obtained, and sample collection was approved by the Ethics Committee of Shanghai Ninth People's Hospital (2016‐167‐T116). The expanded full‐thickness skin specimens were collected from the central area and at least 2 cm from the expansion margin with no surrounding intact skin included. The normal skin specimens were collected independently and were at least 5 cm from the expansion margin (Figure 2A). Masson trichrome staining was performed according to the manufacturer's (Sigma–Aldrich) instructions.

### 2.2 Skin expansion model

The Guide for the Care and Use of Laboratory Animals (US National Institutes of Health, Bethesda, MD, USA) was followed. The study protocol was approved by the Committee on the Ethics of Animal Experiments of Shanghai Jiao Tong University School of Medicine. All animals were housed in individual cages in a specific pathogen‐free (SPF) environment under a controlled temperature (23‐25°C), humidity (45%‐55%) and a 12/12 hours light/dark cycle.

Twenty‐one male Wistar rats (8 weeks old; body weight 250‐270 g; Sino‐British SIPPR/BK Laboratory Animal Co Ltd) were randomly allocated to the expansion group (n = 18) or control group (n = 3). Rats in the expansion group were randomly assigned to six time‐points (n = 3 per time‐point): 1, 2, 4, 24, 96 and 168 hours. The rats were anesthetized with isoflurane (3% for induction and 2% for maintenance). Then, 10‐mL silicone expanders (Shanghai Xin Sheng Biomedical) were implanted subcutaneously on the dorsal sides. Seven days post‐implantation, the expansion procedure was initiated, and the time at which the expansion began was defined as 0 hours. In the expansion groups, 0.9% saline was injected through the pots to expand the tissue expanders and to maintain the intra‐capsular pressure at 60 mm Hg; in the control group, nothing was injected and the expander was left non‐expanded. At each time‐point, full‐thickness skin specimens from the expanded area were collected in the expansion groups. In the control group, specimens from the implanted area were collected at 0 hours. The animals were euthanized immediately after sample collection.

### 2.3 Microarrays

Total RNA was extracted from skin specimens with the TRIzol^®^ Reagent (Invitrogen) and purified further using an RNeasy mini‐kit (Qiagen). RNA samples were hybridized, washed and scanned with GeneChip Rat Exon 1.0 ST Arrays according to the manufacturer's (Affymetrix) instructions. Gene expression was normalized with robust multi‐array averaging in the dataset. Differentially expressed genes were determined by one‐way ANOVA (*P* < .05). All data generated in this study were compliant with minimum information about a microarray experiment.^21^ The complete dataset is available at the Array Express (https://www.ebi.ac.uk/arrayexpress/), Accession No. E‐MTAB‐8304.

### 2.4 Weighted gene coexpression network analysis (WGCNA)

The WGCNA package (R Foundation, Vienna, Austria)^22^ was used to construct coexpression network of differentially expression genes (DEGs) as previously described.^23^ In detail, the pairwise correlation matrix was computed and transformed into the adjacency matrix with the parameter β determined by the pickSoftThreshold function to fit the scale‐free criterion.^24^ Subsequently, we calculated the topologic overlap matrix. Then, the topologic overlap‐based dissimilarity was used as an input for unsupervised hierarchical clustering by employing a dynamic tree‐cutting algorithm.^25^ The branches of the clustering tree were defined as ‘modules’, which included a group of co‐expressed genes and which were summarized according to module eigengenes.^22^, ^26^ Next, we calculated the correlation between stemness and EMT biomarkers and module eigengenes to identify the significant modules related to stemness and EMT. The hub gene (gene that plays a central role in the scale‐free network) located in the most EMT‐related module was selected via a previously described method.^23^ A network of hub gene was also plotted with Cytoscape.^27^

### 2.5 Cell culture

Recombinant human CCN1 protein (rhCCN1) was purchased from PeproTech (Cat No. 120‐25, Rocky Hill, NJ, USA). In Human CCN1 protein, 91.384% of the amino acids was identical to those in rat CCN1 protein. Primary rat keratinocytes were harvested from the dorsal skin of 3‐day‐old Wistar rats and cultured with Keratinocyte Medium (ScienCell) as previously described.^28^ Calcium concentration was 0.09 mmol/L in the medium, and no additional calcium is added in all the experiments. The culture medium is a serum‐free culture medium which supplied with keratinocytes growth supplement (KGS). Cells were not KGS‐starved except for apoptosis assay. Cells were plated at a density of 2 × 10^5^ cells/mL and incubated in a humidified atmosphere at 37°C with 5% CO_2_. For rhCCN1 treatment, primary keratinocytes were treated with 0.2 µg/mL CCN1 protein. The control group was treated with 0.2 µg/mL bovine serum albumin (BSA).

### 2.6 Cell‐stretching assay

Keratinocytes were plated in six‐well, flexible silicone rubber BioFlex™ plates coated with type‐I collagen at 2 × 10^5^ cells/mL. Cyclic mechanical tension with a 0.5‐Hz sinusoidal curve at 10% elongation was applied using an FX‐5000™ Tension Plus unit (Flexcell, Hillsborough, NC, USA). Cells were harvested immediately after mechanical stretching was complete. Control cells were cultured in the same plates in the same incubator without the application of tension.

### 2.7 Cell counting (CCK‐8) assay

Cells were plated in 96‐well plates at 1 × 10^4^ cells per well. Thereafter, the cells were treated with BSA or rhCCN1 and cultured for the following four durations: 2, 24, 48 and 72 hours. Subsequently, 10 μL CCK8 solution (Dojindo Molecular Technologies) was added into each well. After further incubation for 2 hours, the absorbance (450 nm) was measured with microplate absorbance spectrophotometer (Bio‐Rad^®^).

### 2.8 EdU incorporation assay

After BSA or rhCCN1 treatment, the proliferating cells were labelled using an EdU (5‐ethynyl‐2′‐deoxyuridine) DNA Cell Proliferation Kit (RiboBio). After BSA or rhCCN1 treatment, 50 µmol/L EdU was immediately added into the culture medium and incubated for 6 hours. Next, cells were fixed with 4% paraformaldehyde for 20 minutes and then incubated with Apollo Dye Solution for 30 minutes at room temperature to label the proliferating cells. Cell nuclei were stained using Hoechst 33342. The percentage of EdU positive cells was measured with ImageJ (NIH, USA).

### 2.9 *rhCCN1 injection *in vivo

To evaluate the effect of CCN1 on rat normal skin, we randomly assigned ten male adult Wistar rats (8 weeks; body weight 250‐270 g) to the rhCCN1 group (n = 5) or BSA control group (n = 5). The hair was removed, and the dorsal skin was injected intracutaneously with rhCCN1 (2 µg/mL, daily) or BSA (2 µg/mL, daily) as a control. Injection was performed as a single‐spot, and the injected area was outlined. The rats were sacrificed on day 5, and full‐thickness skin specimens were collected from the injected areas for further examination.

To investigate the effect of CCN1 on expanded skin, ten male adult Wistar rats (8 weeks; body weight 250‐270 g) were randomly allocated to the rhCCN1 group (n = 5) or BSA control group (n = 5). In the rhCCN1 group, the entire expanded area was injected with rhCCN1 (0.1 µg/cm^2^, i.c., daily), whereas in the control group, the same amount of BSA (0.1 µg/cm^2^, i.c., daily) was injected. Injection was initiated on expansion day 0. On day 7, ultrasound (GE Healthcare, UK) was used to detect the skin thickness and laser speckle contrast analysis (LASCA) was performed to visualize blood perfusion in the microcirculation.^29^ The perfusion value was calculated using moorFLPI V2.0 (Moor Instruments). The rats were sacrificed on day 7.

### 2.10 Short hairpin RNA (shRNA) knockdown of CCN1 in vivo

To explore the role of CCN1 in expanded skin in vivo, three shRNAs were designed (Table S1) to specifically target rat CCN1 (NM_031327). The plasmids expressing the CCN1 shRNAs were constructed using the pHBAd‐U6 adenovirus vector (Hanbio Biotechnology) and verified by sequencing. pAd‐CCN1 was cotransfected into HEK293T cells for packaging with the skeleton plasmid pHBAd‐BHG to produce the recombinant adenovirus particles. Scrambled shRNA (shNC) was used as a control. For in vivo transfection, 1 × 10^8^ adenovirus particles (200 μL) were injected intracutaneously throughout the entire expansion area at expansion day 0 (n = 5). Rats were sacrificed on day 7.

### 2.11 Immunofluorescence

Cells were washed with phosphate‐buffered saline (PBS) and fixed in methanol for 10 minutes. Skin tissues from humans and rats were fixed with 4% paraformaldehyde for 24 hours, embedded in paraffin, and sectioned (thickness = 5 µm). After fixation, cells or sections were permeabilized and blocked in solution containing 2% BSA, 0.5% TWEEN 20, and PBS (pH 7.3) for 1 hour. Cells and sections were incubated with the primary antibody overnight at 4°C and then visualized using a secondary antibody conjugated with fluorophores that absorbed light at 488 nm or 594 nm (Thermo Fisher Scientific). Nuclei were stained with DAPI (Sigma–Aldrich) for 10 minutes. The primary antibodies used are listed in Appendix S1. Images were acquired by Nikon Ni‐U microscope (Nikon) and analysed by ImageJ.

### 2.12 Quantitative real‐time polymerase chain reaction (qRT‐PCR)

Total RNA was extracted with the TRIzol Reagent (Invitrogen), and the RNA concentration was determined using a NanoDrop™ spectrophotometer (ND‐8000; Thermo Fisher Scientific). RNA samples were reverse‐transcribed into cDNA using an M‐MLV system (Promega Biotech). The primers are listed in Table S2. qRT‐PCR was carried out with SYBR Premix (TaKaRa Biotechnology) in an ABI ViiA7 system (Applied Biosystems). Glyceraldehyde 3‐phosphate dehydrogenase was used as the internal control. The relative mRNA expression was calculated using the 2^−ΔΔCt^ method.^30^

### 2.13 Western blotting

Whole‐cell protein or tissue lysates were prepared with radioimmunoprecipitation assay (RIPA) buffer (Cat No. P0013B, Beyotime Biotechnology) containing a phosphatase inhibitor and protease inhibitor (Roche, Basel, Switzerland). Subcellular proteins were extracted using a subcellular protein fractionation kit (78840; Thermo Fisher Scientific). The protein lysates were loaded into a sodium dodecyl sulphate‐polyacrylamide gel and transferred to polyvinylidene difluoride (PVDF) membranes. The membranes were incubated overnight with the primary antibodies at 4°C, followed by incubation with secondary antibodies (Thermo Scientific) for 1 hour at room temperature. Protein expression was detected using an electrochemiluminescence kit (Tanon Science & Technology), and images of the blots were captured using a chemiluminescence imaging system (6100; Tanon Science & Technology). The primary antibodies used are listed in Appendix S1.

### 2.14 Integrin and β‐catenin pathway inhibitor treatment

Integrin αv inhibitor (Cilengitide, S7077) and β‐catenin pathway inhibitor (XAV‐939, S1180) were purchased from Selleck (Shanghai, China). Keratinocytes were treated with 10 µmol/L Cilengitide, 10 µmol/L XAV‐939 or same volume of DMSO as a control, along with 0.2 µg/mL rhCCN1 for 48 hours for Western blotting.

### 2.15 Statistical analysis

All data are presented as the mean ± SD of triplicate experiments. Western blotting and immunofluorescence results were quantified with ImageJ software and analysed using *t* tests (2 groups) or one‐way ANOVA and multiple comparisons (≥3 groups) with GraphPad Prism 6. Significant differences were defined by a *P*‐value of <.05.

## 3 RESULTS

### 3.1 CCN1 (CYR61) is a central ‘hub’ gene in the network of module genes related to EMT during rat skin expansion

**Figure 1:**
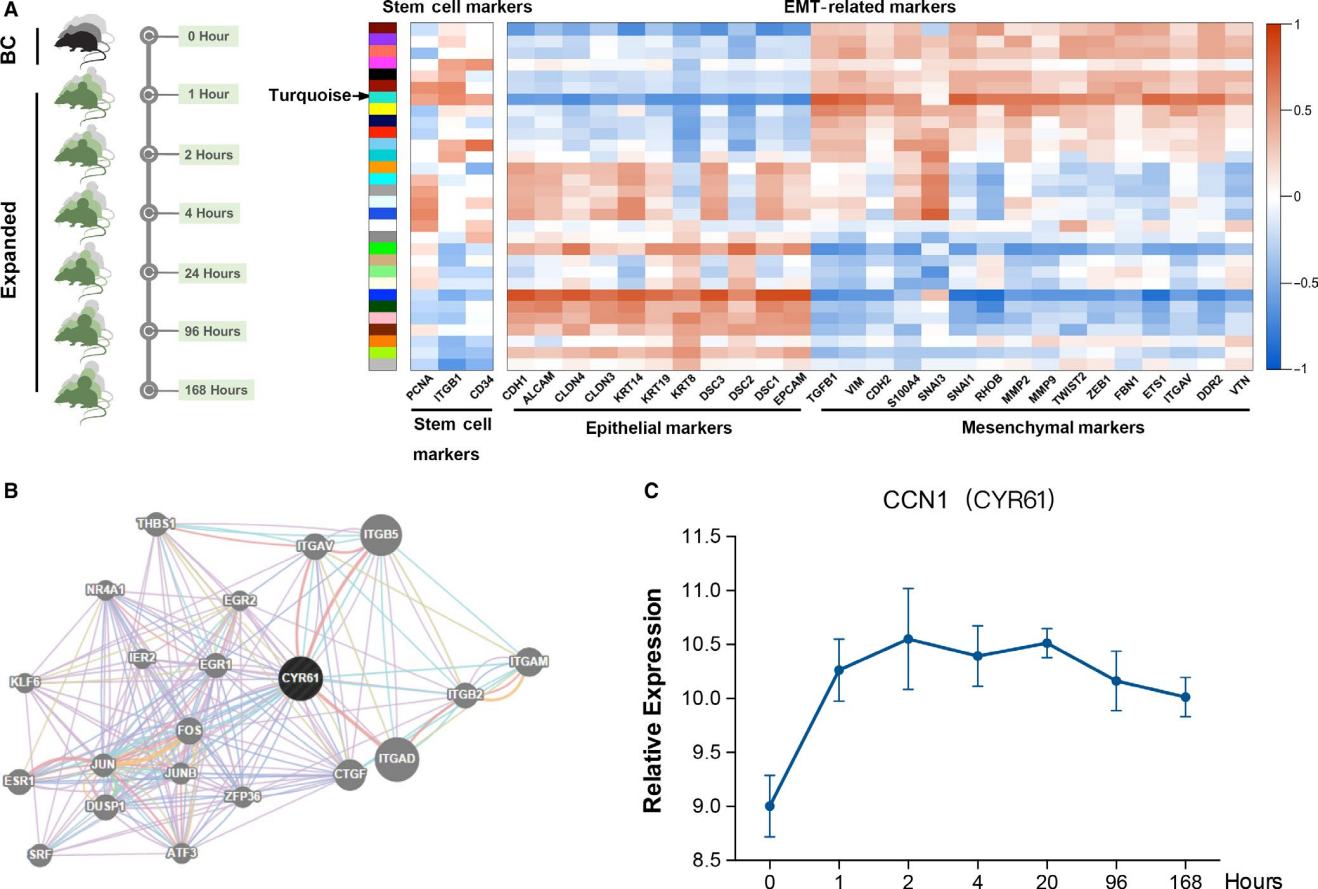
CCN1 (CYR61) is a central ‘hub’ gene in the network of module genes related to EMT during rat skin expansion. A, Skin specimens were collected from rats in the control group (0 h) and expansion groups at six time‐points (1, 2, 4, 20, 96 and 168 h) for the microarray testing (n = 3 per group). The coexpression modules were defined by WGCNA. After associating the coexpression modules with stemness and EMT biomarkers, the most related module was found to be the Turquoise module. B, A network of hub genes was plotted using Cytoscape to visualize the weighed gene coexpression network. CCN1 (CYR61) was found to be the central hub gene in the Turquoise module. C, Time course of the expression of CCN1 (CYR61) generated from the microarray data (n = 3 per group). Values represent the means ± SD

A rat model of skin expansion was established to mimic the clinical process of skin expansion in humans (Figure S1). After analysing the microarrays data by weighted gene coexpression network analysis (WGCNA), we identified the Turquoise module as the most related to stemness and EMT module by correlating module eigengenes with biomarkers of stem cells or EMT (Figure 1A). CCN1 (CYR61) was identified as the top hub gene with a central role in the gene network of the Turquoise module (Figure 1A,B). The time course of expression showed that CCN1 (CYR61) expression was rapidly increased after skin expansion, suggesting that CCN1 could play a crucial role in skin growth after skin expansion (Figure 1C).

### 3.2 CCN1 expression is up‐regulated in human expanded skin

**Figure 2:**
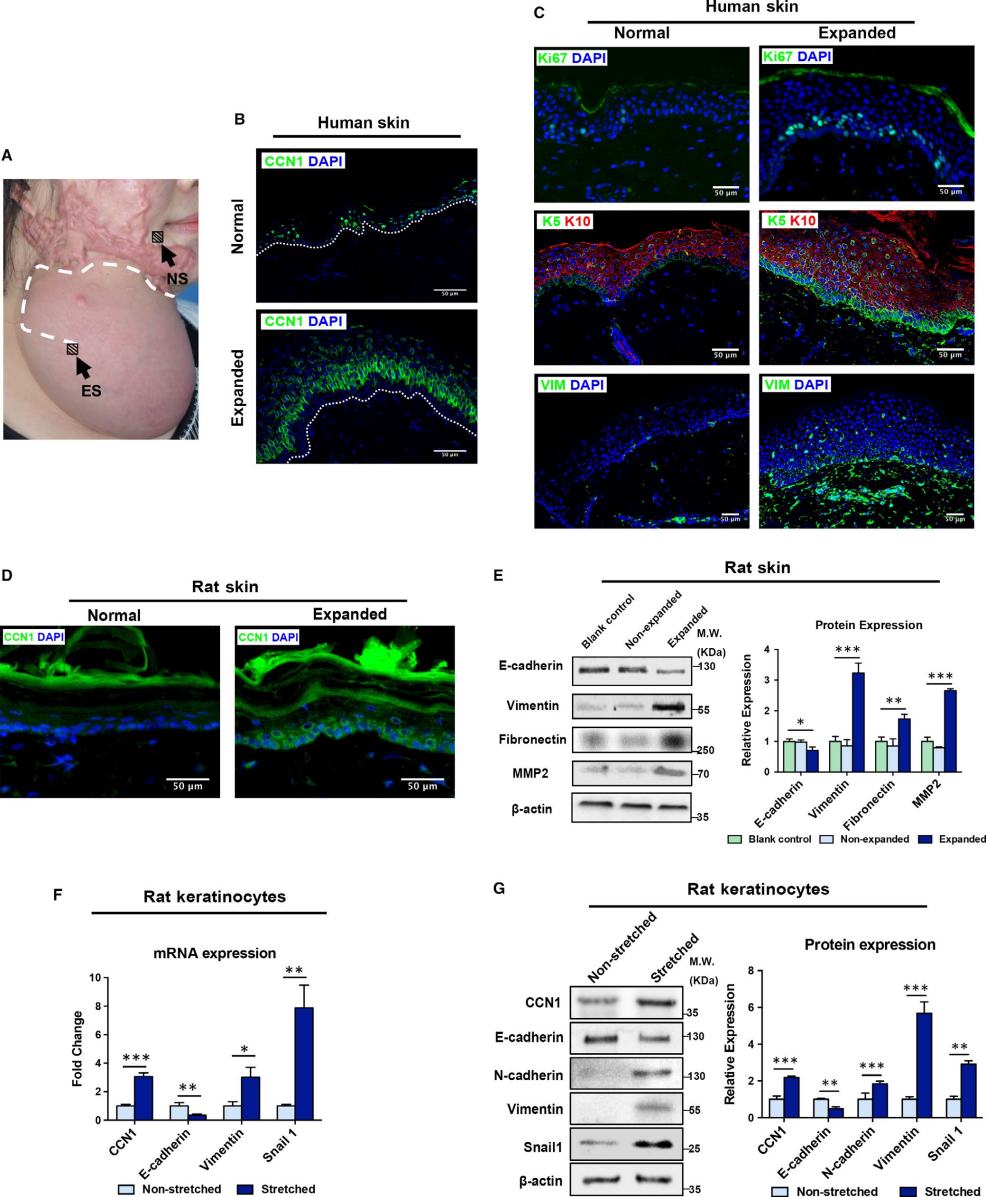
Up‐regulated CCN1 expression is associated with activation of skin growth and enhanced EMT during skin expansion. A, Illustration of sample collection: the white‐dotted line indicated the surgical incision; the black block indicated the sample collection areas for ES (expanded skin) and NS (normal skin). B, Immunofluorescence staining of CCN1 (green) in human normal and expanded skin (n = 10 per group, scale bar: 50 µm). The dotted line indicates the basement membrane. C, Immunofluorescence staining of Ki67 (green), KRT5 (green), KRT10 (red) and Vimentin (green) (n = 10 per group, scale bar: 50 µm). D, Immunofluorescence staining of CCN1 (green) in rat normal and expanded skin (n = 5 per group). E, Western blotting of the EMT markers (E‐cadherin, Vimentin, Fibronectin and MMP2) in the control, non‐expanded and expanded rat skin tissues (left plane). Quantitative analysis of expression of these EMT makers (right panel) (n = 5 per group). F, After a 24‐h of mechanical stretch, qRT‐PCR was conducted for non‐stretched and stretched rat keratinocytes. mRNA expression of CCN1 and EMT markers (E‐cadherin, Vimentin, Snail1) was analysed. (n = 3). G, After a 48‐h of mechanical stretch, Western blotting was conducted for non‐stretched and stretched rat keratinocytes (left plane). Protein expression of CCN1 and EMT markers (E‐cadherin, N‐cadherin, Vimentin, Snail1) was analysed (right panel). (n = 3). All values represent the means ± SD of triplicate determinations. *P < .05, **P < .01, ***P < .001

To detect the expression of CCN1 and visualize the location of CCN1, human expanded skin and adjacent normal skin were collected (Figure 2A) and immunofluorescence staining was performed. The results revealed that CCN1 was mainly positive in the stratum spinosum of the epidermis, specifically in the cytoplasm of keratinocytes and its adjacent ECM. In contrast, CCN1 was barely detectable in the dermis (Figure 2B). Moreover, CCN1 expression was significantly up‐regulated in human expanded skin tissue.

In order to examine the skin growth ability of the expanded skin, we detected the Ki67 level, keratin‐5 (KRT5) level and keratin‐10 (KRT10) level by immunofluorescence staining. The expression of Ki67, which is a marker of proliferation,^31^ in the epidermis of human expanded skin was significantly higher than in that in normal, non‐expanded skin (Figure 2C, top). Usually, KRT5 is a marker of basal keratinocytes and is normally located in the basal layer of the epidermis, whereas KRT10 can be found in suprabasal keratinocytes.^32^ Moreover, part of basal keratinocytes can self‐renew and act as epithelial stem cells that further can differentiate.^33^, ^34^ Our results showed that the number of KRT5‐positive cells was significantly increased and that the number of KRT10‐positive cells was slightly decreased after skin expansion (Figure 2C, middle). Additionally, Vimentin (a mesenchymal marker) expression was significantly up‐regulated in human expanded skin, but relatively low in adjacent normal skin (Figure 2C, bottom). These results suggested that after skin expansion, the epidermis of the expanded skin is in a state of active proliferation; the basal KRT5‐positive keratinocytes proliferate more before they further differentiate to KRT10‐positive keratinocytes; and the mesenchymal phenotype is increased.

### 3.3 Up‐regulated CCN1 expression is associated with enhanced EMT under skin expansion

Similar to human expanded skin samples, elevated CCN1 was observed in the epidermal layer of expanded skin in rats (Figure 2D). We also examined EMT markers in rats by Western blotting. After skin expansion, the expression of E‐cadherin, which is a marker of epithelial cells, decreased, and expression of Vimentin, Fibronectin and matrix metalloproteinase‐2 (MMP2), which are markers of mesenchymal cells, was significantly increased (Figure 2E).

Moreover, we applied a biomechanical force onto keratinocytes to ascertain whether mechanical stretching could result in similar outcome in vitro. Primary cultured keratinocytes were harvested from the epidermis of 3‐day‐old rats, and 10% mechanical stretch was applied. The mRNA levels of CCN1 along with the mesenchymal marker Vimentin and EMT transcriptional factor (EMT‐TF) Snail1 were significantly increased, whereas E‐cadherin expression was significantly decreased (Figure 2F). Similarly, Western blot showed that the protein levels of the CCN1, N‐cadherin, Vimentin and Snail1 proteins were increased, while the expression of E‐cadherin was decreased (Figure 2G).

Overall, our results of both in vivo and in vitro studies demonstrated that skin expansion could up‐regulate the expression of CCN1 as well as acquisition of mesenchymal features.

### 3.4 rhCCN1 treatment directly promotes keratinocyte proliferation

**Figure 3:**
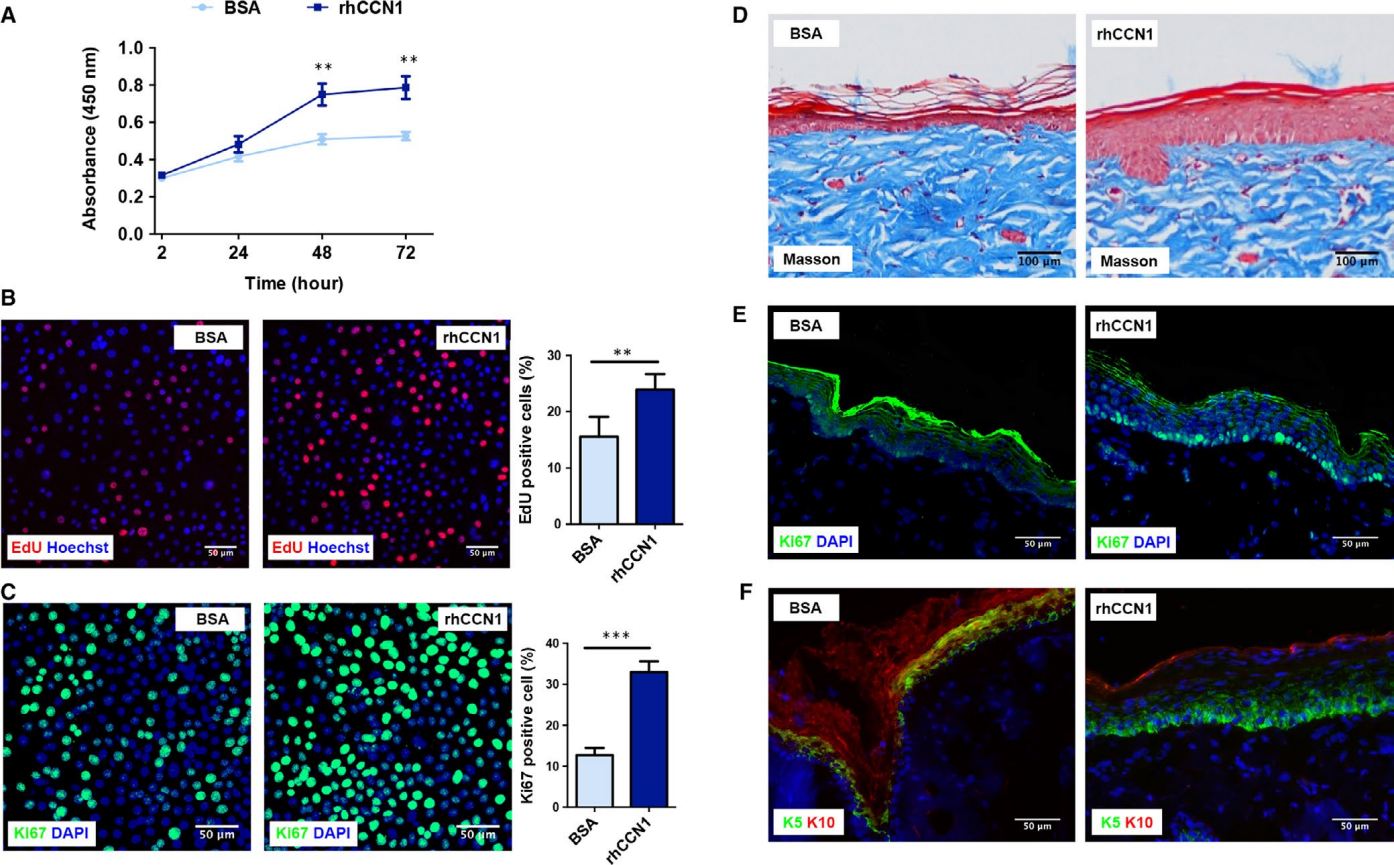
rhCCN1 treatment promotes rat keratinocyte proliferation. A‐C, Primary rat keratinocytes were treated with rhCCN1 (0.2 µg/mL), and BSA (0.2 µg/mL) was used as a control. A, CCK‐8 assays were performed at 2, 24, 48 and 72 h (n = 3). B, EdU staining and quantitative analysis were performed at 48 h (n = 3, scale bar: 50 µm). C, Immunofluorescence staining of Ki67 (green) and quantitative analysis were performed at 48 h (n = 3, scale bar: 50 µm). D‐F, Rat normal skin was subcutaneously injected with rhCCN1 protein for 5 days (2 µg/mL, 50 µL/d), and BSA (2 µg/mL, 50 µL/d) was injected as a control. The rat skin tissues were stained for Masson trichrome (D), Ki67 (green, E), KRT5 (green, F) and KRT10 (red, F) (n = 5 per group, scale bar: 50 µm). All values represent the means ± SD of triplicate determinations. **P < .01, ***P < .001

Subsequently, we assessed the function of the CCN1 protein in vitro. Primary cultured keratinocytes were treated with rhCCN1 (0.2 µg/mL) or BSA control (0.2 µg/mL). The CCK‐8 assay showed that, compared to BSA, rhCCN1 could significantly promote the cell viability of keratinocytes at 48 and 72 hours (Figure 3A). An EdU incorporation assay showed that the DNA synthesis of keratinocytes was significantly enhanced after rhCCN1 stimulation (Figure 3B). Furthermore, our results showed that after rhCCN1 treatment, the number of Ki67‐positive cells was significantly increased (Figure 3C). Additionally, cell apoptosis was examined with propidium iodide (PI)/annexin V through flow cytometry, and the results showed that the extent of early apoptosis of keratinocytes was decreased after CCN1 treatment (Figure S2a). A lower proportion of TUNEL‐positive cells was noted after rhCCN1 treatment (Figure S2b). Taken together, these results indicated that the CCN1 protein could promote keratinocytes proliferation and prevent apoptosis.

To explore the effect of CCN1 on skin tissue in vivo, we injected rhCCN1 (2 µg/mL daily) into the normal skin of rats. On day 5 after rhCCN1 injection, Masson trichrome staining showed that CCN1 treatment increased epidermal thickness compared with the control group (Figure 3D). Moreover, the number of Ki67‐positive cells was significantly increased after rhCCN1 treatment (Figure 3E). Additionally, KRT5 expression was up‐regulated and KRT10 expression was down‐regulated in the epidermis (Figure 3F), indicating that CCN1 could directly induce the proliferation of the basal KRT5‐positive keratinocytes and thus promote the epidermal proliferation ability.

### 3.5 rhCCN1 treatment directly induces partial EMT

**Figure 4:**
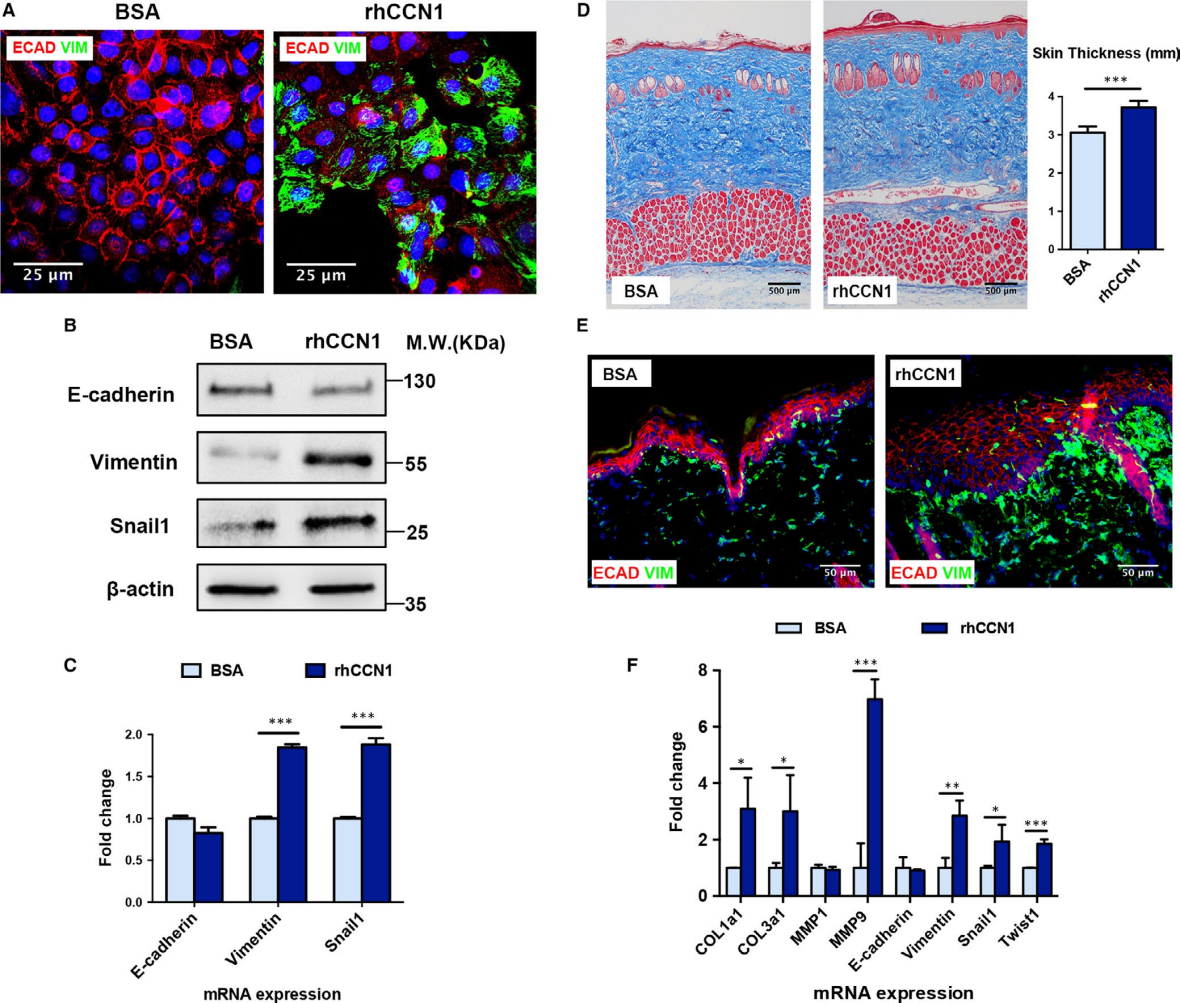
rhCCN1 treatment induces partial EMT in rat keratinocytes and rat normal skin. A‐B, Primary keratinocytes were treated with rhCCN1 (0.2 µg/mL) for 48 h, and BSA (0.2 µg/mL) was used as a control. A, Immunostaining of the epithelial marker E‐cadherin (green) and mesenchymal marker Vimentin (red) (n = 3, scale bar: 25 µm). B, Western blotting of E‐cadherin, Vimentin and Snail1 (n = 3). C, Primary keratinocytes were treated with rhCCN1 (0.2 µg/mL) for 24 h. qRT‐PCR analysis of E‐cadherin, Vimentin and Snail1 (n = 3). D‐F, Rat normal skin was subcutaneously injected with rhCCN1 protein (2 µg/mL, 50 µL/d), and BSA (2 µg/mL, 50 µL/d) was injected as a control. D, Masson trichrome staining of the injected rat skin tissues in both groups (n = 5 per group, scale bar: 100 µm). E, Immunostaining of E‐cadherin (red) and Vimentin (green) in the injected rat skin tissues from both groups (n = 5 per group, scale bar: 50 µm). F, qRT‐PCR analysis of collagen production (COL1a1, COL3a1 and MMP1) and EMT markers (E‐cadherin, Vimentin, Snail1, Twist1 and MMP9) in the injected skin tissues from both groups (n = 5 per group). All values represent the means ± SD of triplicate determinations. *P < .05, **P < .01, ***P < .001

We further explored the relationship between CCN1 and EMT. Keratinocytes were stimulated with CCN1 protein (0.2 µg/mL) for 48 hours. Figure 4A shows a group of rat keratinocytes undergoing partial EMT upon rhCCN1 treatment. Treated keratinocytes lost the epithelial phenotype and simultaneously acquired the mesenchymal phenotype. Western blotting analysis demonstrated that, after treatment with rhCCN1, keratinocytes expressed elevated levels of Vimentin and Snail1 and a reduced level of E‐cadherin compared with the BSA control (Figure 4B). E‐cadherin mRNA levels showed a slight decrease after rhCCN1 treatment, but the levels of Vimentin mRNA and Snail1 mRNA were significantly increased (Figure 4C). These results indicated that CCN1 can act as an inducer of partial EMT in keratinocytes.

To determine whether CCN1 could enhance EMT in vivo, we examined the expression of EMT markers of the rat normal skin injected with rhCCN1. Further analysis showed that the total skin thickness was slightly thickened upon rhCCN1 injection (Figure 4D) and that CCN1‐treated skin exhibited a low content of E‐cadherin and a high content of Vimentin (Figure 4E). qRT‐PCR results showed that collagen production was stimulated and that the expression levels of mesenchymal markers such as Vimentin and matrix metalloproteinase‐9 (MMP9) and EMT‐TFs Snail1 and Twist1 were increased after rhCCN1 treatment (Figure 4F). Taken together, these data demonstrated that, without mechanical stretching, CCN1 could directly induce partial EMT and promote skin growth.

### 3.6 CCN1 induces partial EMT via β‐catenin pathway

**Figure 5:**
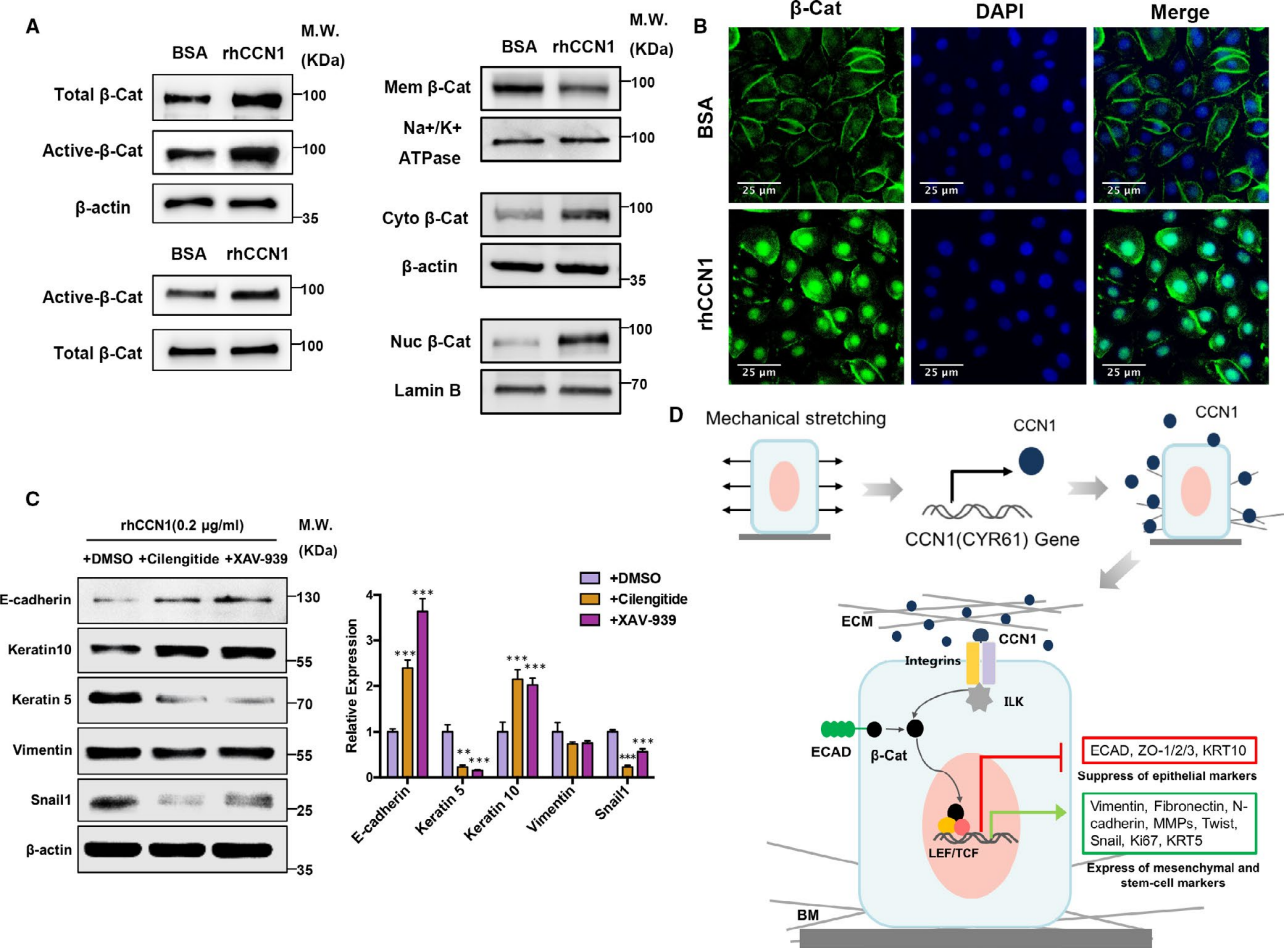
CCN1 induces partial EMT via β‐catenin pathway. A‐B, Primary keratinocytes were treated with rhCCN1 (0.2 µg/mL) for 48 h, and BSA (0.2 µg/mL) was used as a control. A, Western blotting detected the β‐catenin pathway activation after rhCCN1 treatment (left panel). Western blotting of lysates after subcellular fractionation showed the expression of β‐catenin of cell membrane, cytoplasm and nucleus (right panel). (n = 3) B, Cells were stained for β‐catenin (green) and DAPI (blue) after rhCCN1 treatment (n = 3). Scale bar: 50 µm. C, CCN1‐treated keratinocytes were treated with the integrin inhibitor (Cilengitide) or the β‐catenin inhibitor (XAV‐939), and DMSO was used as a control (n = 3). Calcium concentration was 0.09 mmol/L in the medium, and no additional calcium was added. Western blotting was then performed to examine the expression of E‐cadherin, KRT10, KRT5, Vimentin and Snail1 (left panel). Right panel showed the quantitative results of Western blotting. Values represent the means ± SD of triplicate determinations. **P < .01, ***P < .001. D, CCN1 expression and signal transduction after mechanical stretching. Schematic diagram: mechanical stretching increases the production of CCN1, which binds to integrins, leading to integrin‐linked kinase (ILK) activation and stimulation of β‐catenin signalling by promoting β‐catenin translocation, thus triggering the expression of downstream EMT‐associated genes, which finally enhances EMT

The β‐catenin pathway is reportedly related to EMT.^35^, ^36^ Here, we found that the β‐catenin pathway was activated after rhCCN1 stimulation on keratinocytes. As shown in Figure 5A (left), the levels of total β‐catenin and active β‐catenin were increased after rhCCN1 treatment. Additionally, the level of active β‐catenin was found to be elevated after the level of total β‐catenin was normalized. Moreover, Western blotting of subcellular protein fractions showed that membrane β‐catenin was decreased whereas cytoplasmic and nuclear β‐catenin were increased after rhCCN1 treatment (Figure 5A, right). Similarly, IF staining of β‐catenin showed the same results, indicating that β‐catenin translocated from the cell membrane to the cytoplasm and cell nucleus upon rhCCN1 treatment (Figure 5B). Since CCN1 is reported to be an integrin α‐v ligand and integrin αv is required for keratinocyte proliferation,^37^, ^38^ we also investigated whether this rhCCN1‐induced partial EMT could be inhibited by an integrin αv inhibitor (Cilengitide) or a β‐catenin pathway inhibitor (XAV‐939). Western blotting showed that both inhibitors could reverse the effect of rhCCN1, increasing the expression of E‐cadherin and KRT10 while decreasing the expression of KRT5, vimentin and Snail1 compared with the DMSO control (Figure 5C). Taken together, these findings suggest that CCN1 can induce partial EMT by activating the β‐catenin pathway, initiating β‐catenin translocation and thus trigger the expression of downstream genes (Figure 5D).

### 3.7 rhCCN1 injection promotes skin growth and improves the efficiency of skin expansion in rats

**Figure 6:**
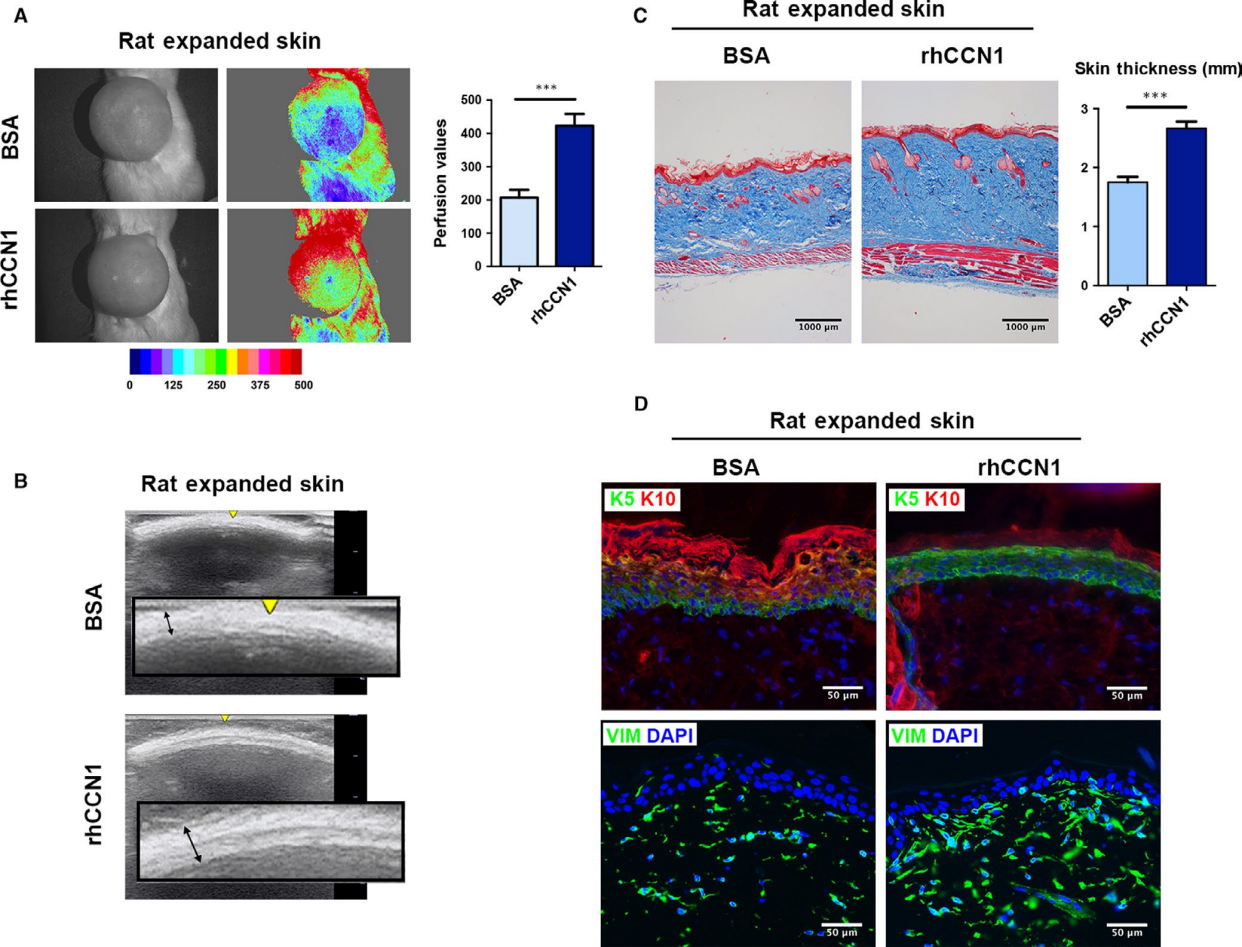
rhCCN1 injection promotes the skin growth and improves the efficiency of skin expansion. Rat skin was expanded as described in the Methods, and the entire expanded area was injected with rhCCN1 (0.1 µg/cm^2^, i.c., daily) or same amount of BSA (0.1 µg/cm^2^, i.c., daily). The rats were sacrificed on day 7. A, Skin tissue blood perfusion was analysed by laser speckle contrast analysis (LASCA) on day 7 (n = 5 per group). B, Ultrasound test on day 7 (n = 5 per group). C, Masson trichrome staining of the expanded skin tissues from both groups (n = 5 per group, scale bar: 1000 µm). Skin thickness was measured by Image J. D, Immunostaining of KRT5 (green), KRT10 (red) and Vimentin (green) of the expanded skin tissues from both groups (n = 5 per group, scale bar: 50 µm). All values represent the means ± SD of triplicate determinations. ***P < .001

To investigate the therapeutic effect of CCN1 on skin expansion in vivo, we established a model of skin expansion in rats (Figure S1) and the entire expanded area was injected with rhCCN1 (0.1 µg/cm^2^, i.c., daily) or same amount of BSA (0.1 µg/cm^2^, i.c., daily). On day 7, the rhCCN1‐treated group exhibited a better blood supply than the BSA control group according to the results of laser speckle and ultrasound (Figure 6A,B). Masson trichrome staining also showed that the total thickness of the expanded flaps was increased in rhCCN1‐treated rats (increased by 52.3%, *P* < .001) compared with control rats. We further observed dermal thickening and increased amount of collagen fibres in the rhCCN1‐treated group compared with that in the control group (Figure 6C).

Furthermore, immunofluorescence of the expanded skin showed dramatically increased expression of KRT5 and reduced expression of KRT10 in rhCCN1‐treated rats than that of BSA‐treated rats, suggesting the occurrence of further proliferation of basal keratinocytes upon rhCCN1 treatment under skin expansion (Figure 6D, top). Additionally, Vimentin expression in CCN1‐treated rats was significantly higher than that in BSA‐treated rats, indicating that the mesenchymal traits were further enhanced upon rhCCN1 treatment under skin expansion (Figure 6D, bottom). Taken together, these results indicate that CCN1 can promote skin growth and improve the efficiency of skin expansion by enhancing EMT in vivo, and it has a high translational value in clinical practice.

### 3.8 CCN1 knockdown by shRNA suppresses the skin growth and reduces the efficiency of skin expansion

**Figure 7:**
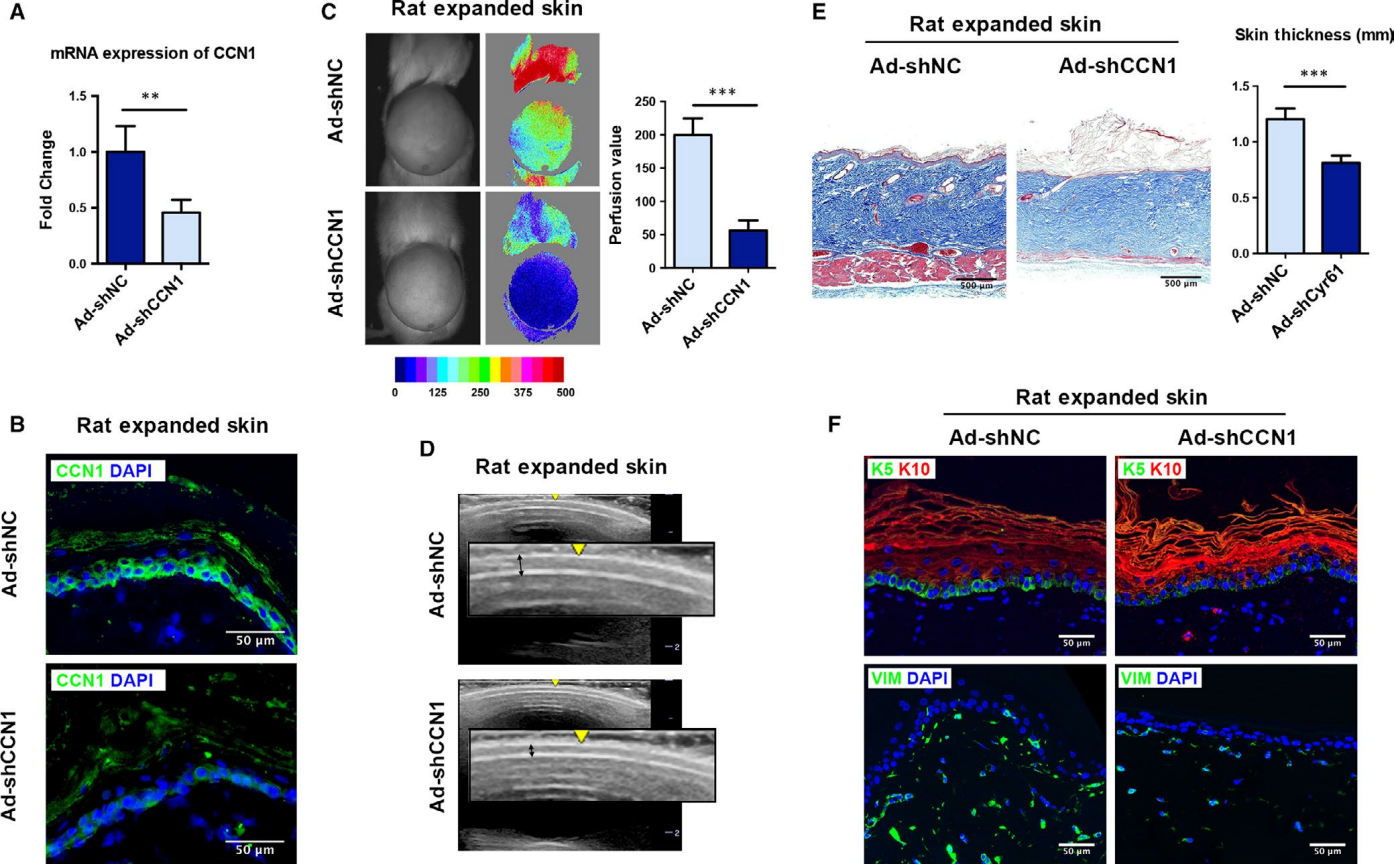
shRNA knockdown of CCN1 suppresses the skin growth and reduces the efficiency of skin expansion. Rat expanded skin was transfected with Ad‐shNC (control shRNA) or Ad‐shCCN1 (CCN1‐targeting shRNA). The rats were sacrificed on day 7. qRT‐PCR (A) and immunostaining (B) of CCN1 were performed to confirm knockdown of CCN1 in the rat skin (n = 5 per group, scale bar: 50 µm). C, Blood perfusion of the expanded skin was analysed by LASCA on day 7 (n = 5 per group). D, Ultrasound examination was performed on day 7 (n = 5 per group). E, Masson trichrome staining of the expanded skin tissues from both groups (n = 5 per group, scale bar: 500 µm). Skin thickness was measured by Image J. F, Immunostaining of KRT5 (green), KRT10 (red) and Vimentin (green) in the expanded skin tissues from both groups (n = 5 per group, scale bar: 50 µm). All values represent the means ± SD of triplicate determinations. **P < .01, ***P < .001

To investigate the effect of CCN1‐inhibiting on skin expansion in vivo, we intracutaneously injected three shRNAs targeting CCN1 into a rat model. After qRT‐PCR examination, the Ad‐shCCN1‐S1 was determined to have the highest knockdown efficiency and designated Ad‐shCCN1 in the following experiments (Figure S3). The qRT‐PCR and immunofluorescence results verified that the Ad‐shCCN1 could successfully knockdown CCN1 expression in expanded skin by 54.2% (*P* = .002, Figure 7A,B). The haemodynamics and ultrasound images of the Ad‐shNC group and Ad‐shCCN1 group were recorded on day 7. The Ad‐shCCN1‐treated group showed a significantly poorer blood supply and decreased flap thickness compared with the Ad‐shNC group (Figure 7C,D). Masson trichrome staining also showed the expanded flaps of the Ad‐shCCN1 group were significantly decreased by 32.7% (*P* < .001) in flap thickness compared with the Ad‐NC group (Figure 7E). Besides, decreased dermal thickness and amount of collagen fibres were observed in the Ad‐shCCN1 group compared with that in the control group (Figure 7E).

Immunofluorescence staining showed significantly lower KRT5 and higher KRT10 expression in the Ad‐shCCN1 group, suggesting less proliferation of basal keratinocytes upon CCN1 silencing (Figure 7F, top). The mesenchymal traits were reduced after CCN1 knockdown as the Vimentin expression was decreased in Ad‐shCCN1 group (Figure 7F, bottom). These results confirm that CCN1 acts as a crucial player in promoting EMT and skin growth during skin expansion.

## 4 DISCUSSION

Epithelial‐mesenchymal transition (EMT) has been demonstrated to be a crucial process in various developmental events and tissue regeneration.^39^ EMT is characterized by loss of the epithelial phenotype, with reduced expression of the cell adhesion protein (eg E‐cadherin), and an acquired mesenchymal phenotype accompanied by increased expression of (eg Vimentin).^16^ E‐cadherin is a well recognized epithelial marker as lack of E‐cadherin results in the loss all epithelial phenotype and the dissociation of β‐catenin from cell junctions, and Vimentin is a reliable mesenchymal marker.^40^ The key of EMT is the transcriptional factors (EMT‐TFs) Snail and Twist,^41^, ^42^ which modulate the whole process. In the current study, by WGCNA analysis of microarray data generated from rat expanded skin, we found that CCN1 is the hub gene in the network of module genes related to EMT.

CCN1 (CYR61) is an immediate‐early response gene and a dynamically expressed matricellular protein that interacts with various integrins on cell membranes.^10^ In addition to the transcriptional activation of CCN1 by various growth factors and cytokines,^43^ CCN1 expression is sensitive to a wide range of extracellular stimuli, especially mechanical stimuli.^44^, ^45^, ^46^ It has been reported that CCN1 expression can be induced by mechanical stretching in both vascular and bladder smooth muscle cells.^46^, ^47^ However, the effect of CCN1 in mechanically stretched skin tissue has not been studied. In the present study, we first found that the CCN1 protein is elevated in expanded human and rat skin, indicating that CCN1 is a mechanical sensitive protein that might play a potent role in skin expansion. Moreover, previous studies have revealed that CCN1 is a target gene of the Yes‐associated protein (YAP) signalling^48^ and our microarray data also showed that YAP is a early response transcriptional factor after skin expansion (data not shown). Therefore, YAP signalling may exert its effect via CCN1 during skin expansion. Further series of studies involving YAP‐CCN1 and skin growth are anticipated to investigate a detailed gene function network during skin expansion.

CCN1 has been recognized to exert multiple potent functions in the proliferation, survival, migration, angiogenesis, apoptosis and senescence of cells.^49^ Previous studies have shown that CCN1 plays a critical role in promoting wound healing, tissue repair and restricting fibrosis.^50^, ^51^, ^52^ Sun et al^53^ demonstrated that CCN1 could promote keratinocyte activation and was involved in psoriatic pathogenesis. Our recent work revealed that CCN1 could improve cutaneous healing by accelerating reepithelialization and promoting keratinocytes migration.^54^ Similarly, the current study demonstrated that CCN1 protein could stimulate keratinocytes proliferation and prevent early cell apoptosis.

Furthermore, our in vitro and in vivo studies revealed that the up‐regulation of CCN1 protein corresponding to mechanical stretch is associated with enhanced EMT. The relationship between CCN1 and EMT remains a major question. CCN1 has been reported to induce EMT in gastric epithelial cells.^55^ It is widely accepted that epithelial cells that have undergone EMT or partial EMT lose their apical‐basal polarity; exhibit altered interaction with the ECM; gain a mesenchymal phenotype, characterized by enhanced proliferation and migration abilities^16^, ^56^; and gained stemness traits.^18^ Several studies have demonstrated that keratinocytes that have undergone EMT can migrate into the wound bed.^57^, ^58^ Here, we found that CCN1 could directly induce keratinocytes to gain mesenchymal traits and up‐regulate EMT‐TFs (eg Snail, Twist) as well as increased proliferation in vitro and in vivo*.* Accordingly, we conclude that CCN1 could stimulate skin growth by initiating partial EMT. Moreover, apart from epidermis thickening, we also observed increased dermal thickness and enhanced collagen production in CCN1‐treated skin tissue. Thus, we hypothesized that part of the CCN1‐induced EMT cells may migrate to the dermis and become mesenchymal‐like cells to produce collagen and contribute to extracellular matrix remodelling during skin expansion. From this perspective, further research involving lineage tracing is necessary to evaluate the proportion of partial EMT cells and their dynamic changes during skin expansion to support our conclusion.

The β‐catenin signalling pathway is one of the major signalling pathways in EMT process.^16^, ^41^ Previous studies have indicated that the binding of CCN1 to integrins leads to integrin‐linked kinase (ILK) activation, stimulates β‐catenin signalling^59^ and thus promotes the transcriptional activation of downstream target genes, including EMT‐associated genes.^60^ Here, we revealed that CCN1 can activate the β‐catenin signalling pathway and induces nuclear translocation of β‐catenin in keratinocytes. Additionally, our results demonstrated that both an integrin αv inhibitor and β‐catenin inhibitor can reverse CCN1‐induced partial EMT and reduced proliferation of basal keratinocytes. Therefore, we concluded that the binding of CCN1 to integrin αv could activate the β‐catenin pathway and thus enhance EMT which eventually promoted skin growth.

Finally, we investigated the effect of CCN1 protein on skin expansion in a rat model. Our results demonstrated that CCN1 administration during skin expansion could further increase the flap thickness, enhanced the proliferation of basal keratinocytes and induce partial EMT of the expanded skin. In contrast, the inhibition of CCN1 with shRNA interference could result in a thin, poorly vascularized flap, restrict the growth ability and reduced EMT. These results suggested that CCN1 is a crucial enhancer of skin growth and has a high translational value for clinical practice. The major studies focusing on improving the efficiency of skin expansion include stem cell therapy (eg BM‐MSCs, BM‐MNCs and ADSCs),^5^, ^61^, ^62^ growth factors therapy (eg bFGF)^63^ and others treatments (eg botulinum toxin A, tanshinon IIA).^64^, ^65^ While, our recent work showed that CCN1 is superior to bFGF^54^ in accelerating wound healing. Further studies are needed to compare the therapeutic effects of CCN1, stem cell therapy and growth factors on promoting skin growth.

In summary, our study demonstrates that CCN1(CYR61) is a crucial actor in skin expansion and that CCN1 can promote skin growth by enhancing EMT via the β‐catenin pathway. Moreover, intracutaneous injection of rhCCN1 promotes skin growth during skin expansion. If applicable to in humans, CCN1 could be a potential therapeutic target for promoting skin growth and improving the efficiency of skin expansion in clinical practice.

## CONFLICT OF INTEREST

The authors declare that they have no conflicts of interests.

## AUTHOR CONTRIBUTIONS

Yiwen Zhou carried out the main part of the studies and drafted the manuscript. Haizhou Li and Xiao Liang revised the manuscript and contributed to data curation. Hengyu Du and Yinjun Suo conducted the human sample collection and data processing. Hao Chen helped with the study design and performed the statistical analysis. Wenhui Liu contributed to implementation of the computer code. Ran Duan contributed to the animal model establishing. Xiaolu Huang developed and executed the idea of identifying hub genes during skin expansion. Qingfeng Li supervised the project and contributed to funding acquisition.

## Data Availability

The raw data of microarray that support the findings of this study are openly available in Array Express at https://www.ebi.ac.uk/arrayexpress/, Accession No. E‐MTAB‐8304.
