# Supplementary material for: The CCN1 (CYR61) protein promotes skin growth by enhancing epithelial‐mesenchymal transition during skin expansion
Source: J Cell Mol Med. 2019 Dec 11;24(2):1460–73. doi: 10.1111/jcmm.14828 (PMC6991652; doi:10.1111/jcmm.14828)
Supplement: Supplementary file 1 [file JCMM-24-1460-s001.docx]

**Supplementary Materials**

**Supplementary Methods**

*Apoptosis assay*

Keratinocytes were seeded onto six-well plates and treated with 0.2 µg/mL rhCCN1 or 0.2 µg/mL BSA as a control. Keratinocytes were keratinocyte growth supplement (KGS) starved for 4 hours before rhCCN1 treatment. After 48 hours, the cells were harvested. Apoptosis was measured using annexin V-fluorescein isothiocyanate/PI double-staining (Beyotime Biotechnology, Shanghai, China) followed by flow cytometry (FACSCalibur™, Becton Dickinson, Franklin Lakes, NJ, USA). The results were expressed and calculated using CellQuest™ (BD Biosciences). TUNEL staining was performed with the TUNEL Apoptosis Detection Kit (Yeasen Biotech, Shanghai, China) according to the manufacturer’s instructions. Cell nuclei were labelled with 4',6-diamidino-2-phenylindole (DAPI). The percentage of TUNEL positive cells was measured by ImageJ.

*The primary antibodies used in immunofluorescence*

CCN1 (1:100 dilution; [sc-13100](https://www.scbt.com/scbt/product/cyr61-antibody-h-78?requestFrom=search), Santa Cruz Biotechnology, Santa Cruz, CA, USA), Ki67 (1:150; AB9260, Millipore, Bedford, MA, USA), KRT5 (1:200; ab52635, Abcam, Cambridge, UK), KRT10 (1:200; ab9025, Abcam, Cambridge, UK), Vimentin (1:200; ab92547, Abcam, Cambridge, UK) and β-catenin (1:200; A10834, Abclonal, Wuhan, China).

*The primary antibodies used in western blotting*

CCN1 (1:500 dilution; [sc-13100](https://www.scbt.com/scbt/product/cyr61-antibody-h-78?requestFrom=search), Santa Cruz Biotechnology, Santa Cruz, CA, USA), E-cadherin (1:500; 610181, BD Biosciences), Fibronectin (1:500; ab2413, Abcam, Cambridge, UK), KRT5 (1:500; ab52635, Abcam, Cambridge, UK), KRT10 (1:500; ab76318, Abcam, Cambridge, UK), Lamin B (1:500; sc-374015, Santa Cruz Biotechnology, Santa Cruz, CA, USA), MMP2 (1:500; ab37150, Abcam, Cambridge, UK), Na+-K+ATPase (1:500; sc-28800, Santa Cruz Biotechnology, Danvers, MA, USA), N-cadherin (1:500; ab18203, Abcam, Cambridge, UK), Snail1 (1:500; ab53519, Abcam, Cambridge, UK), total β-catenin (1:1000; 8480, Cell Signaling Technology, Danvers, MA, USA), active-β-catenin (1:1000; 8814, Cell Signaling Technology, Danvers, MA, USA), Vimentin (1:500; ab8978, Abcam, Cambridge, UK), β-actin (1:1000; 4970, Cell Signaling Technology, Danvers, MA, USA).

**Supplementary Figures**


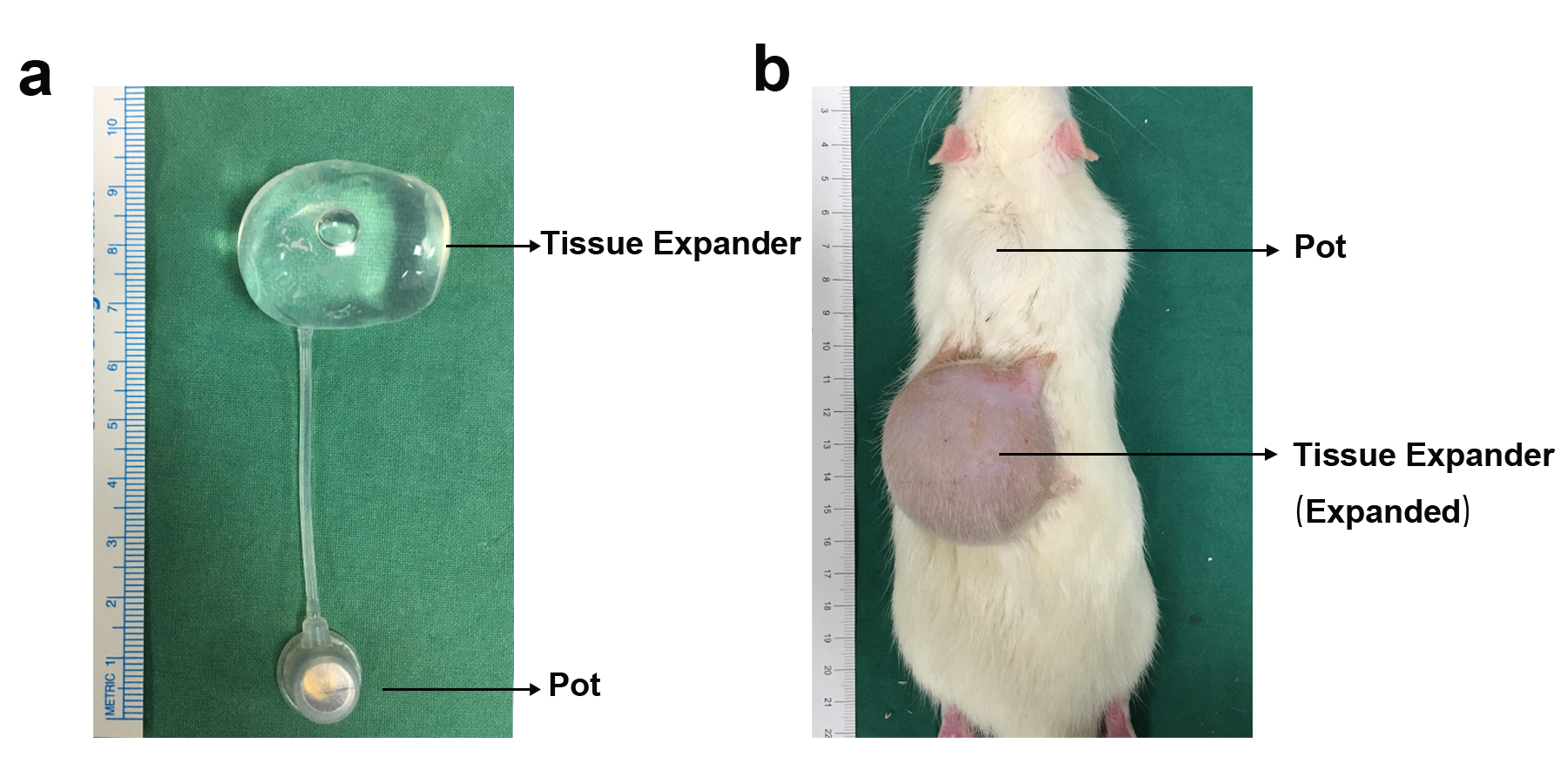


***Figure S1. Model of skin expansion in rats.*** *(****a****) Silicone tissue expander and its application. The tissue expander is a balloon-like silicone implant with a pot. (****b****) Saline can be injected through the pot to enlarge the expander.*

**
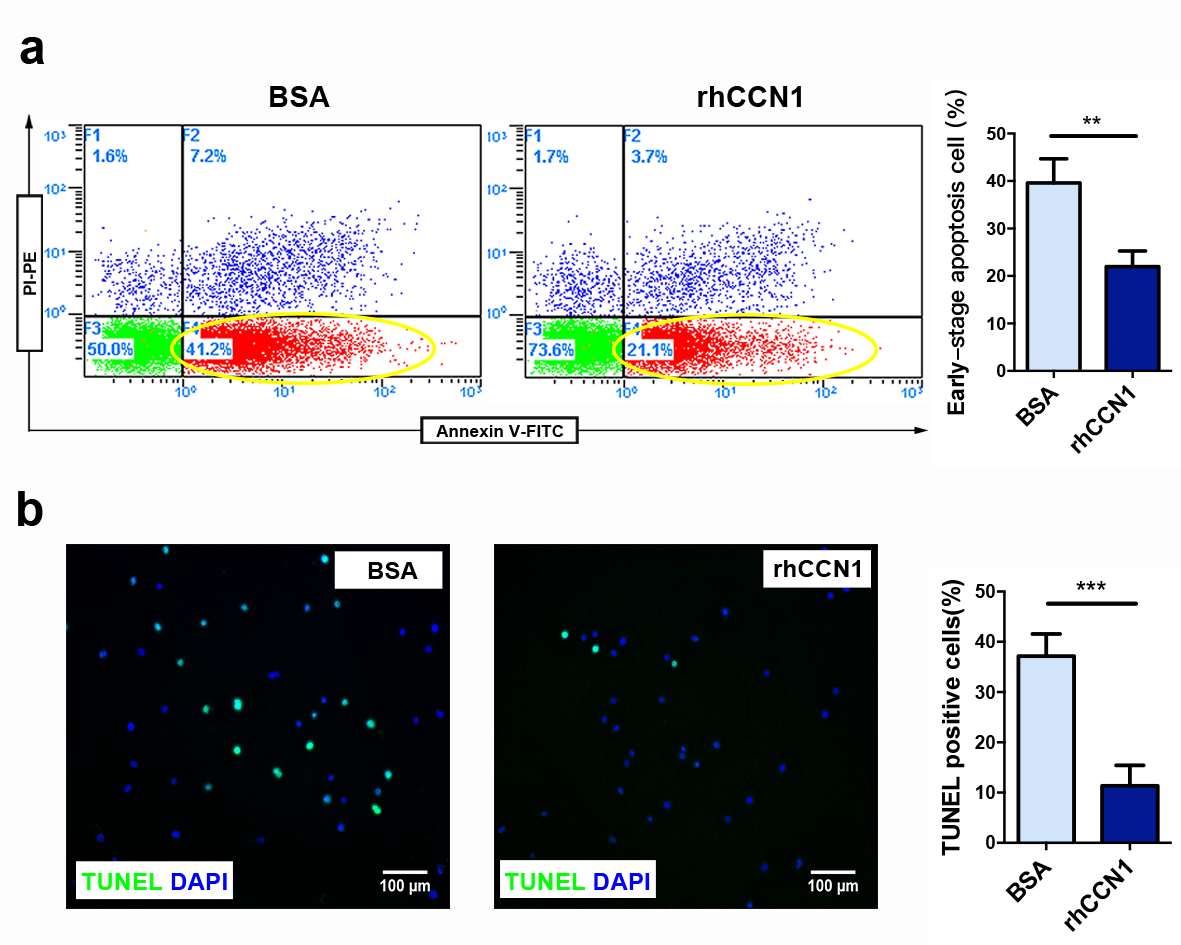
**

***Figure S2.*** ***Cell apoptosis was reduced after CCN1 treatment.*** ***(a-b)*** *Primary keratinocytes were KGS-starved for 4 hours before treated with rhCCN1 (0.2 µg/mL) or BSA control (0.2 µg/mL) for 48 hours.* ***(a)*** *Apoptosis was examined by PI/annexin V double-staining through flow cytometry. The percentage of early-stage apoptosis cells was counted (yellow circle). Left panel shows the quantitative analysis in rhCCN1-treated keratinocytes (21·97%) compared with the control group (39.60%). (n = 3.)* ***(b)*** *TUNEL staining was performed to exam cell apoptosis (left panel). The percentage of TUNEL-positive cells were quantified by Image J (n = 3): 11.4% TUNEL-positive cells in rhCCN1 group; 37.2% TUNEL-positive cells in BSA control group. Scale bar: 100 µm.* *All values represent the means ± SD of triplicate determinations. **p<0.01, ***p<0.001.*

*
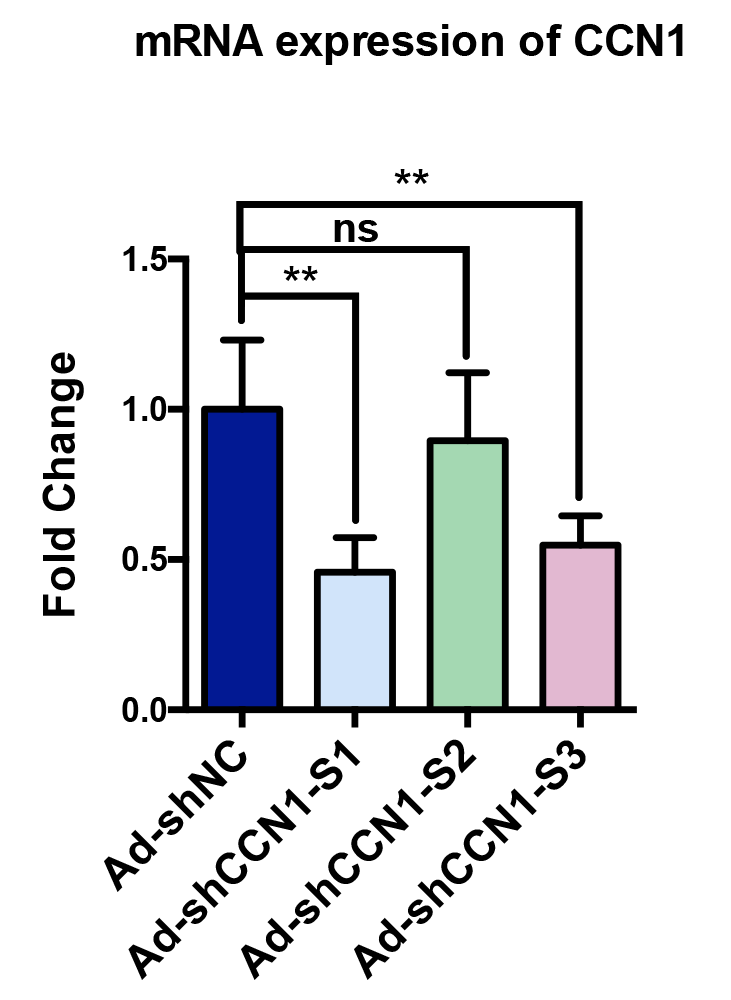
*

***Figure S3. The shRNA screening.*** *All three shRNAs targeting CCN1 were transfected to rat expanded skin on day0. CCN1 expression was detected by qPCR at day 7. The knockdown efficiency is 54.2% for Ad-shCCN1-S1, 10.4% for Ad-ShCCN1-S2, and 45.1% for Ad-ShCCN1-S3 (n = 5). Ad-shCCN1-S1 was selected and named as Ad-shCCN1 for further experiments. ns: non-significant, **p<0.01.*

**Supplementary Tables**

| shRNA |  | Oligonucleotide sequence |
| --- | --- | --- |
| shCCN1-S1 | Sense  Anti-sense | 5'AATTCGCCCATGGCCAGAAATGCATCGTTCATTCAAGAGATGAACGATGCATTTCTGGCCATGGGTTTTTTG  5'GATCCAAAAAACCCATGGCCAGAAATGCATCGTTCATCTCTTGAATGAACGATGCATTTCTGGCCATGGGCG3' |
| shCCN1-S2 | Sense  Anti-sense | 5'AATTCGATCTGTGAAGTGCGTCCTTGTGGATTCAAGAGATCCACAAGGACGCACTTCACAGATCTTTTTTG 3'  5'GATCCAAAAAAGATCTGTGAAGTGCGTCCTTGTGGATCTCTTGAATCCACAAGGACGCACTTCACAGATCG 3' |
| shCCN1-S3 | Sense  Anti-sense | 5'AATTCGCGCCTCTACAGTCTGTTCAACGATATTCAAGAGATATCGTTGAACAGACTGTAGAGGCGTTTTTTG 3'  5'GATCCAAAAAACGCCTCTACAGTCTGTTCAACGATATCTCTTGAATATCGTTGAACAGACTGTAGAGGCGCG 3' |
| shNC | Sense  Anti-sense | 5'AATTCGTTCTCCGAACGTGTCACGTAATTCAAGAGATTACGTGACACGTTCGGAGAATTTTTTG3'  5'GATCCAAAAAATTCTCCGAACGTGTCACGTAATCTCTTGAATTACGTGACACGTTCGGAGAACG3' |

***Table S1. The sequences of shCCN1s and shNC are listed.***

| Gene | Primers | |
| --- | --- | --- |
| CCN1 | forward | 5' ATCTGTGAAGTGCGTCCTTGT 3' |
|  | reverse | 5' TCTGGGGATTTCTTGGTCTTG 3' |
| E-cadherin | forward | 5' CCAACAGGGACAAAGAGACAA 3' |
|  | reverse | 5' CAATGATGAAAACGCCAACA 3' |
| Snail1 | forward | 5' GGCTGATGGAAGGCAGAGT 3' |
|  | reverse | 5' TGGGTTGGCTTTAGTTCTATGG 3' |
| Vimentin | forward | 5' AAAGCAGGAGTCAAACGAATAC 3' |
|  | reverse | 5' CTCTTCCATTTCACGCATCT 3' |

***Table S2. The target genes and corresponding primers are listed.***
